# Analysis of Transitional Milk of Post-Natal Cases in Non-Anaemic Mothers and Its Comparison With Anaemic Mothers in Rural Western Uttar Pradesh

**DOI:** 10.7759/cureus.11818

**Published:** 2020-12-01

**Authors:** Pragati Divedi, Parul Maheshwari, Shikha Seth, Archana Mishra

**Affiliations:** 1 Obstetrics and Gynaecology, Santosh Medical College and Hospital, Ghaziabad, IND; 2 Obstetrics and Gynaecology, Uttar Pradesh University of Medical Sciences, Saifai, IND; 3 Obstetrics and Gynaecology, Government Institute of Medical Sciences​, Greater Noida, IND; 4 Obstetrics and Gynaecology, Vardhman Mahavir Medical College and Safdarjung Hospital, New Delhi, IND

**Keywords:** anaemic mothers, non-anaemic mothers, post-natal, transitional milk

## Abstract

Aim: Present study is aimed at determining the analysis of transitional milk of post-natal non-anaemic mothers and its comparison with anaemic mothers in rural Uttar Pradesh.

Methods: Totally, 132 post-natal cases were enrolled for the study. After taking ethical committee approval, breast milk samples were collected from day 4 to 11. We measured the following important parameters in breast milk (fat, density, carbohydrates, solid not fat [SNF], protein and added water). Data were analysed by using SPSS-24 software (IBM Corp., Armonk, NY). Tests used in our study were the analysis of variance (ANOVA) test, Chi-square test and independent T-test.

Result: In our study, it was found that severe anaemia causes significant changes in fat, lactose and protein content of breast milk. We found that there are no significant changes in breast milk composition with age. Our study shows statistically no association between residence and breast milk content.

Conclusion: As the severity of anaemia increases, protein and fat content in breast milk decreases, lactose content on the contrary follows a reverse relationship with maternal haemoglobin. Maternal anaemia not only affects the macronutrients in breast milk but also decreases the density of breast milk.

## Introduction

The benefits of breastfeeding, provide protection for the health of the infant during the first weeks of life. These are short- or medium-term effects: a highly protective effect on infant mortality, with a 12% decrease in mortality risk compared to non-breastfed [1]; a decrease in respiratory and gastrointestinal infections during the first weeks of life of the newborn [2], probably related to the composition of the colostrum (immature milk for the first three days of life) and breast milk that confers immune protection to the child. Finally, there is also a consensus on the effect of breastfeeding on the improvement of neurodevelopment [3], in premature babies [4], or on the term [5]. The first fluid produced by mothers after delivery (for one to four days after birth) is colostrum, rich in immunologic components and there is a transitional milk phase from 8 to 20 days which happens between the colostrum and mature milk phase. The mature milk phase starts around 20 days after birth. Analysis of human milk revealed that it has two components (macronutrients and micronutrients). The macronutrients are mainly proteins, fat and lactose. The most abundant proteins are casein, α-lactalbumin, lactoferrin, secretory immunoglobulin IgA, lysozyme and serum albumin. Human milk fat is characterized by high contents of palmitic and oleic acid. Fat is the most highly variable macronutrient of milk. The principal sugar of human milk is the disaccharide lactose. Many micronutrients vary in human milk depending on maternal diet and body stores, including vitamins A, B1, B2, B6, B12, D, and iodine. The richness of breast milk in miRNA is also one of its characteristics [6,7]. miRNAs are non-coding RNAs that regulate gene expression and control protein synthesis at the post-transcription level. A haemoglobin level of less than 11 mg/dl is considered as anaemia in pregnancy and postpartum. The level of haemoglobin used for classification of anaemia in pregnant women as mild, moderate and severe are those recommended by the Indian Council of Medical Research (ICMR) as follows: mild anaemia (Hb 10.0-10.9 mg/dl), moderate anaemia (Hb 7.0-10.0 mg/dl), severe anaemia (Hb 4-7.0 mg/dl), very severe anaemia (Hb less than 4.0 mg/dl). As in the rural population, it is difficult to get postpartum women back to the hospital again after 1 month for mature milk examination, we have planned our study on transitional milk composition. We planned to analyse the major nutrients and components of transitional milk in anaemic mothers of various severities to know how it is different from the milk of healthy non-anaemic mothers.

## Materials and methods

This study was conducted in the Department of Obstetrics and Gynaecology, UPUMS, Saifai, Etawah, Uttar Pradesh, India over a period of 18 months from January 2018 to June 2019. This study was conducted in compliance with the ethical standards of the responsible institution on human subjects as well as with the Helsinki Declaration. After taking institutional ethical committee approval, totally 132 cases were enrolled for the study out of which 66 cases belonged to anaemic group (group 1 with haemoglobin less than 11 g/dl) and 66 cases to non-anaemic group (group 2 with haemoglobin more than 11 g/dl) based on their antenatal haemoglobin status after checking the inclusion and exclusion criteria.

Inclusion criteria were: (i) post-natal clients were divided into anaemic and non-anaemic group based on haemoglobin status in the antenatal period at the time of their admission; (ii) post-natal clients with term deliveries; (iii) post-natal clients on days 4 to 11; (iv) all postpartum women aged 18-40 years of age; (v) all cases with singleton pregnancies. Exclusion criteria include: (i) patients with a history of blood transfusion within the last one month; (ii) patients with certain medical conditions like hypertensive disorders, tuberculosis, diabetes, thyroid disorders, leukemia and bleeding diathesis, chronic renal disease, human immunodeficiency virus (HIV) and patients on chemotherapy; (iii) post-natal patients with preterm deliveries; (iv) post-natal cases with multiple pregnancies; (v) post-intra-uterine death (IUD) deliveries; (vi) post-natal cases with a history of postpartum haemorrhage (PPH) following delivery.

After counselling, the cases and taking proper written and informed consent, breast milk samples (20-30 mL) were collected from postpartum patients from days 4 to 11. Samples were collected in the morning one hour after the previous breastfeeding. Breast milk was expressed by hand or manual pump and collected into plastic containers. The collected samples were immediately instilled in the milk analyser available in the hospital setup. No preservatives were added. Milk analyser (Model-Julie Z9 FULMATIC, ScopeElectric, Razgrad, Bulgaria) is a fully automatic milk analyser. It measures the following important parameters in the breast milk: fat, density, carbohydrate, solid not fat (SNF), protein, added water. Data were analysed by using SPSS-24 software (IBM Corp., Armonk, NY). Tests used in our study were (analysis of variance, ANOVA, test, Chi-square test, independent T-test).

## Results

Table 1 shows the distribution of cases according to their age, parity and residence. The majority of cases in the study belong to the age group 21-30 years (81.2% in anaemic and 78.8% in non-anaemic) and the rest were between age group 31-40 years (6.1% in anaemic and 6.1% in non-anaemic). The mean age of anaemic group taken understudy was 25.27±3.32 years and for the non-anaemic group was 25.12±4.12 with a p-value of 0.8182 (statistically nonsignificant). In this table, we can see that majority of cases belong to para-4 in anaemic group of women (30.3%) followed by para-2 (25.7%) in contrast to the non-anaemic group, in which most cases were primigravida (42.4%). The mean parity of the anaemic group was 2.74±1.17, while that of the non-anaemic group was 2.30±1.31 with a p-value of 0.0439 (statistically significant). This suggests that the distribution of cases according to parity was unequal and the majority of cases included in anaemic group were multipara as compared to the non-anaemic group. In this table, we can see that the total number of anaemic women in the rural population was found to be 49 out of 95 (51.6%). The total number of anaemic women in the urban population was 17 out of 37 (46%). Anaemia was to be more in the rural population, but in our study, this difference was statistically non-significant with a p-value of 0.561.

**Table 1:** Distribution of cases according to age, parity and residence in anaemic and non-anaemic mothers.

	Anaemic N (%)	Non-anaemic N (%)	Total	P-value
Age				
≤20 years	8 (12.1%)	10 (15.1%)	18	
21-30 years	54 (81.2%)	52 (78.8%)	106	0.8182
31-40 years	4 (6.1%)	4 (6.1%)	8	
Total	66	66	132	
Mean ± SD	25.27±3.32	25.12±4.12		
Parity				
1	10 (15.1%)	28 (42.4%)	38	
2	17 (25.7%)	6 (9.1%)	23	0.0439
3	16 (24.2%)	20 (30.0%)	36	
4	20 (30.3%)	9 (13.6%)	29	
˃4	3 (4.5%)	3 (4.5%)	6	
Total	66	66	132	
Mean ± SD	2.74±1.17	2.30±1.31		
Residence				
Urban	17 (25.7%)	20 (30.3%)	37 (28.0%)	0.561
Rural	49 (74.2%)	46 (69.7%)	95 (72%)	
Total	66	66	132	

Table 2 shows a comparison of density and SNF of breast milk between anaemic and non-anaemic mothers. According to the table mean density of breast milk in the case of anaemic mothers was 23.67 g/cm^3^ while in non-anaemic mothers it was 24.28 g/cm^3^. The table suggests that maternal anaemia not only affects the macronutrients but also the density of breast milk (p-value 0.007). In this table, we can see that the mean SNF content in anaemic mothers is 7.02±0.27% and in non-anaemic mothers is 6.92±0.38% with a p-value of 0.84, which is statistically insignificant.

**Table 2:** Shows comparison of density and SNF of breast milk between anaemic and non-anaemic mothers. SNF: solid not fat

	Mean (%)	Standard deviation (%)	P-value
Density			
Group 1 (anaemic)	23.67 g/cm^3^	0.91 g/cm^3^	0.00
Group 2 (non-anaemic)	24.28 g/cm^3^	1.57 g/cm^3^	
SNF			
Group 1 (anaemic)	7.02	0.27	0.084
Group 2 (non-anaemic)	6.92	0.38	

Table 3 shows a comparison between breast milk content of non-anaemic and anaemic (mild, moderate, and severe) mothers. According to this study, the mean fat content in non-anaemic mothers is 5.59%, whereas, in the severely anaemic group, it is 5.10% with a p-value of 0.0001 (statistically highly significant). In moderately anaemic mothers mean fat content is 5.32% with a p-value of 0.0166 (statistically significant). In mildly anaemic mothers, it is 5.49% with a p-value of 0.6473 (statistically non-significant). The mean lactose content of non-anaemic mothers is 5.63% and in severely anaemic mothers is 6.21% with a p-value of 0.0001 (statistically highly significant). Mean lactose in moderately anaemic mothers is 6.17% with a p-value of 0.0001 (statistically highly significant). Mean lactose in mildly anaemic mothers is 6.05% with a p-value of 0.0003, which is significant. Mean protein content in non-anaemic mothers is 1.18% and in severely anaemic mothers is 0.74% with a p-value of 0.0001 (statistically highly significant). Mean protein content in moderately anaemic mothers is 0.89% with a p-value of 0.0001 (statistically highly significant). Mean protein content in mildly anaemic mothers is 1.08% with a p-value of 0.0001 (statistically highly significant). This table suggests that severe anaemia causes significant changes in fat, lactose and protein content of breast milk, moderate anaemia also causes significant changes in fat, lactose and protein contents of milk while mild anaemia has some effect on lactose and protein contents of breast milk but not on fat content.

**Table 3:** Shows comparison of breast milk contents between non-anaemic and anaemic (mild, moderate, severe) mothers.

	Fat		Lactose		Protein	
	Mean ± SD	P-value	Mean ± SD	P-value	Mean ± SD	P-value
Status of anaemia						
Non-anaemic (n=66)	5.59±0.54	0.0001	5.63±0.41	0.0001	1.18±0.15	0.0001
Severely anaemic (n=25)	5.10±1.37		6.21±0.57		0.74±0.10	
Non-anaemic (n=66)	5.59±0.54	0.0166	5.63±0.41	0.0001	1.18±0.15	0.0001
Moderately anaemic (n=35)	5.32±1.52		6.17±0.83		0.89±0.13	
Non-anaemic (n=66)	5.59±0.5367	0.64786	5.63±0.4113	0.0003	1.18±0.1540	0.0001
Mildly anaemic (n=6)	5.49±1.4048		6.05±0.7010		1.08±0.2053	

Table 4 shows the association between age and fat, lactose and protein content in breast milk. Mean fat values in mothers with age less than 30 years is 5.41%, and that of women with age more than 30 years is 5.52% with a p-value of 0.66, which is statistically insignificant. Mean lactose content in mothers with age less than 30 years is 5.91%, and that of women with age more than 30 years is 5.91% with a p-value of 0.56, which is statistically insignificant. The mean protein content of mothers with age less than 30 years is 1.02%, and that of women with age more than 30 years is 1.03%, with a p-value of 0.947 which is statistically insignificant. This table depicts that there are no significant changes in breast milk composition with age.

**Table 4:** Association between age and fat, lactose and protein contents in breast milk.

Variables	Age (years)	N	Mean (%)	Standard deviation	Standard error mean	T	P-value
Fat	<30	124	5.41	1.1545	0.1037	0.441	0.660
	˃30	8	5.52	0.6159	0.2177		
Lactose	<30	124	5.91	0.6369	0.0572	0.585	0.560
	˃30	8	5.91	0.7070	0.2500		
Protein	<30	124	1.02	0.2315	0.0208	0.066	0.947
	˃30	8	1.03	0.2765	0.0977		

Table 5 shows the association between parity and fat, lactose and protein contents in breast milk. This table shows that there is no association between the increase in parity with the breast milk composition.

**Table 5:** Association between parity and fat, lactose and protein contents in breast milk.

Variables	Parity - 1, 2, 3, 4, ˃4	Sum of squares	Mean square	F	P-value
Fat	Between groups	7.333	1.467	1.159	0.333
Lactose	Between groups	1.653	0.331	0.803	0.550
Protein	Between groups	0.517	0.103	1.969	0.088

Table 6 shows a comparison of fat, lactose and protein content in breast milk between rural and urban populations. According to this table, the mean fat content in the rural population was 5.43±1.20% while in the urban population was 5.46±0.93%, with a p-value of 0.631 which is statistically insignificant. Mean lactose content in the rural population and the urban population was 5.93±0.66% and 5.85±0.60%, respectively, with a p-value of 0.631, which is statistically insignificant. Mean protein content in rural and urban populations was 1.02±0.23% and 1.01±0.23% with a p-value of 1.00, which is statistically insignificant. According to this table, there is statistically no association between residence and breast milk content.

**Table 6:** Comparison of fat, lactose and protein contents in breast milk between rural and urban population.

Factors	Group	Mean±SD (%)	t-value	P-value
Fat	Rural (n=95)	5.43±1.20	0.481	0.631
	Urban (n=37)	5.46±0.93		
Lactose	Rural (n=95)	5.93±0.66	0.481	0.631
	Urban (n=37)	5.85±0.60		
Protein	Rural (n=95)	1.02±0.23	0.0	1.000
	Urban (n=37)	1.01±0.23		

## Discussion

In western Uttar Pradesh, India, especially rural areas early marriage and childbearing are common. Repeated and frequent pregnancies and deliveries that are not covered by medical advice, lead to a progressive increase in the burden of anaemia despite so many health policies by the government. In the present study, the majority of cases belonged to the age group of 21 to 30 years (81.8% in anaemic group and 78.7% in non-anaemic group) followed by less than 20 years (12.1% in anaemic and 15.1% in non-anaemic group). The least number of cases belonged to 31-40 years of age (6% in anaemic and 6% in non-anaemic group). The study conducted by Rajamouli et al. [8], showed similar results, in which 59.1% belonged to the age group of 20-24 years. The least number of cases belonged to age more than 30 years (0.7%). Another study conducted by Gebre and Mulugeta showed similar results with majority of subjects between the age group of 18 and 24 years (40.3%) [9]. In our study, most of the anaemic antenatal women enrolled for study were multigravida (30.3% belonged to para-4) whereas in the non-anaemic group most of the women were primigravida (42.4%). The least number of women were grand multipara (4.5% in anaemic and 4.5% in non-anaemic group) because of the overall reduction in fertility in our country. Similar results were observed in a study conducted by Rajamouli et al., in which the majority of anaemic cases belonged to para 2 (43.3%), whereas in non-anaemic cases the majority of cases were primigravida (32.7%) [8]. Socio-economic status is an important factor for anaemia. In our study, the majority of cases belonged to rural areas (74.2% in anaemic and 69.6% in non-anaemic group). Similar results were also found by Rajamouli et al. [8], in which women suffering from anaemia in classes 1 and 2 were less as compared with lower, lower-middle and middle-socio-economic status (94.11%, 94.49% and 93.51%), respectively. Mean fat content in the breast milk of severely anaemic mothers was 5.10%, in moderately anaemic mothers was 5.32% and mildly anaemic ones was 5.49%, which was less than the mean fat content of non-anaemic mothers (5.59%) with a p-value of 0.0001, which is statistically highly significant. With the increase in severity of anaemia, the fat content in breast milk increases. The result of the study was similar to the study conducted by Corbitt et al. [10], in which maternal anaemia was associated with significantly lower milk fat content (p-value 0.025). The mean lactose content of severely anaemic mothers was 6.21%, in moderately anaemic mothers was 6.17%, in mildly anaemic was 6.05%, which was more than that of non-anaemic mothers (5.63%) with a p-value 0.0001 which is statistically significant. In contrast, the study conducted by Corbitt et al. [10], showed that there was no significant effect of maternal anaemia on lactose content in breast milk. Our study showed that the mean protein content in the breast milk of anaemic mothers (0.85%) was less than that of non-anaemic mothers (1.18%) with a p-value of 0.0001, which is statistically significant. The result of our study was in contrast to the study conducted by Corbitt et al. [10], in which maternal anaemia was associated with a significant increase in protein content in breast milk (p-value 0.001). In our study, we found that changes in fat, lactose, and protein contents with age are insignificant. In contrast, a study conducted by Ronit Lubetzky et al. [11], showed that fat content in colostrum and carbohydrate content in mature milk were significantly higher in the older mother groups. A similar study conducted by Wu Xinali et al. [12] showed that protein concentration was highest in the milk of mothers who are 20-30 years old and carbohydrate content in mature milk was significantly higher in the older mother groups. In our study, breast milk content is not affected by parity. In contrast, the study conducted by Czosnykowska-Lukacka et al. [13] stated that in the group of women with one child, the levels of total protein in breast milk were significantly lower compared to women with two children. In contrast, the concentration of carbohydrates was higher in the milk of mothers having the first child. In our study, we found that breast milk contents are not affected by residence. Similar results were found in a study conducted by Butts et al. [14], where breast milk nutrient profiles of women from different ethnicities were similar in their macronutrient composition.

## Conclusions

In our study, we found that, as the severity of anaemia increases, protein and fat contents in breast milk decrease, lactose content in the contrary follows a reverse relationship with maternal haemoglobin. Maternal anaemia not only affects the macronutrients in breast milk but also decreases the density of breast milk at a significant level. We found that maternal age does not affect the quality of breast milk in terms of fat, lactose and protein. Quality of breast milk is not affected by parity and residence of the women.
